# Malignant pleural mesothelioma metastatic to the submandibular salivary gland, simulating glandular hypertrophy, diagnosed by fine-needle aspiration biopsy: a case report and literature review

**DOI:** 10.1186/1477-7819-12-129

**Published:** 2014-04-28

**Authors:** Massimo Ambroggi, Elena Orlandi, Raoul P Foroni, Luigi Cavanna

**Affiliations:** 1 Department of Oncology and Hematology, Oncology Unit, Azienda Ospedaliera ‘Guglielmo da Saliceto’, Via Taverna 49, 29100 Piacenza, Italy; 2 Department of Pathology, Azienda Ospedaliera ‘Guglielmo da Saliceto’, Via Taverna 49, 29100 Piacenza, Italy

**Keywords:** Mesothelioma, Salivary gland metastasis, Fine needle aspiration

## Abstract

**Background:**

Malignant mesothelioma is a rare neoplasm that generally develops in the pleural or peritoneal cavity. Distant metastases are common; it rarely metastatizes to the head and neck region.

**Case presentation:**

A 54-year-old white man, a non-smoker, was treated with chemotherapy, surgery and radiation for a malignant pleural mesothelioma. Seven months after the last treatment, he developed a right submandibular enlargement: clinical examination, ultrasound and computerized tomography scans revealed a salivary gland hypertrophy. Anti-inflammatory and antibiotic treatment was then started, without improvement. An ultrasound (US)-guided fine-needle aspiration biopsy (FNAB) showed atypical mesothelial cells with nuclear enlargement and increased chromatin representation. Immunocytochemistry showed positivity for calretinin and WT-1.

A diagnosis of right submandibular salivary gland involvement from mesothelioma was established, allowing an adequate treatment.

**Conclusion:**

We report a very rare site of metastasis from malignant pleural mesothelioma. We suggest that US-guided FNAB is a useful, quick, and cheap procedure for a definite diagnosis.

## Background

Malignant mesothelioma is a rare neoplasm with an incidence rate of 1 per 100,000 (both sexes, United States) [1].

Approximately 80% of cases of mesothelioma develop in the pleural cavity, whereas the other 20% arise in the peritoneum; the pericardium is affected very rarely [2].

Metastases from malignant mesothelioma are common, usually to the local lymph nodes, pleurae of the contralateral lung, bone, liver and brain [3]. However, metastases to the head and neck region are uncommon but the jaw bones, particularly the mandibular molar area, are more likely to be the site of metastases than the perioral region [4-19].

We present a 54-year-old man, a non-smoker, with no occupational exposure to asbestos with malignant pleural mesothelioma, previously treated with surgery, radiotherapy and chemotherapy. He presented with right submandibular salivary gland enlargement, diagnosed using US and computerized tomography (CT) scan imaging techniques, as salivary gland hypertrophy, while a fine-needle aspiration biopsy (FNAB) showed metastasis from malignant mesothelioma.

## Case presentation

A 54-year-old Caucasian man underwent right pleural biopsy in December 2005 for recurrent pleural effusion and locally advanced malignant mesothelioma was diagnosed. Subsequently, he was treated with chemotherapy containing cisplatin and pemetrexed for seven cycles; CT scan showed a partial response. In December 2006, he underwent surgery involving total right pleuro-pneumonectomy plus partial resection of the pericardium and diaphragm. The histological features showed epithelioid malignant mesothelioma involving the parietal and the visceral pleurae, the lung, the pericardium and the diaphragm.

Subsequent radiotherapy to the right chest was administered using 50.4 Gy, 28 fractions.

The patient remained disease-free until June 2009 when an isoechogenic and hypodense nodule of 35 × 16 mm was discovered on US and CT scan in the right side of the anterior abdominal wall. An ultrasound-guided biopsy showed metastasis of mesothelioma. In August 2009, the nodule was surgically removed, and radiotherapy was then administered to this site using 45 Gy, 15 fractions.

In September 2011, a relapse in the mediastinum (lymph nodes) was found by CT scan and Positron Emission Tomography (PET), so six cycles of chemotherapy with cisplatin and pemetrexed were started which lasted until January 2012. Complete response at PET examination in February 2012 was demonstrated.

**Figure 1:**
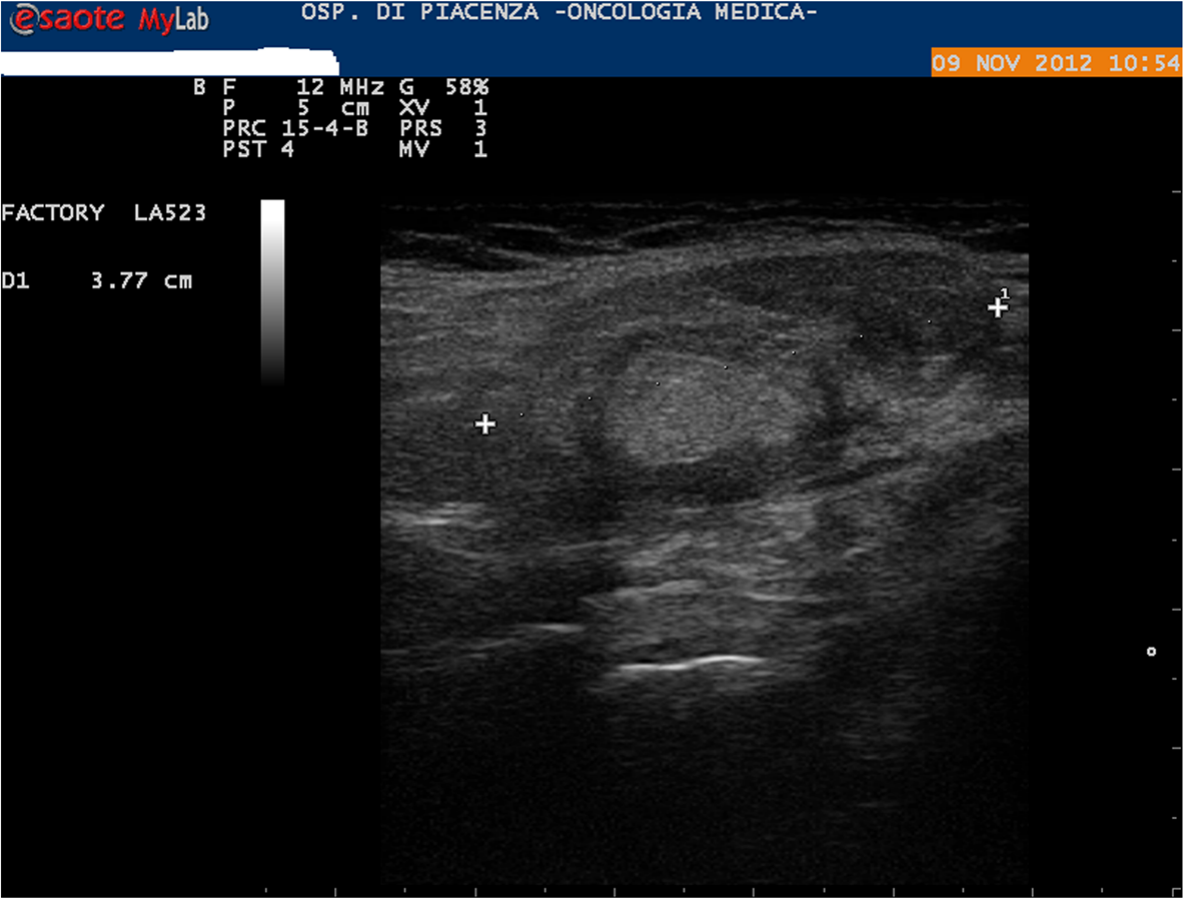
Ultrasound image showing a right submandibular salivary gland enlargement with a low-echogenic pattern.

**Figure 2:**
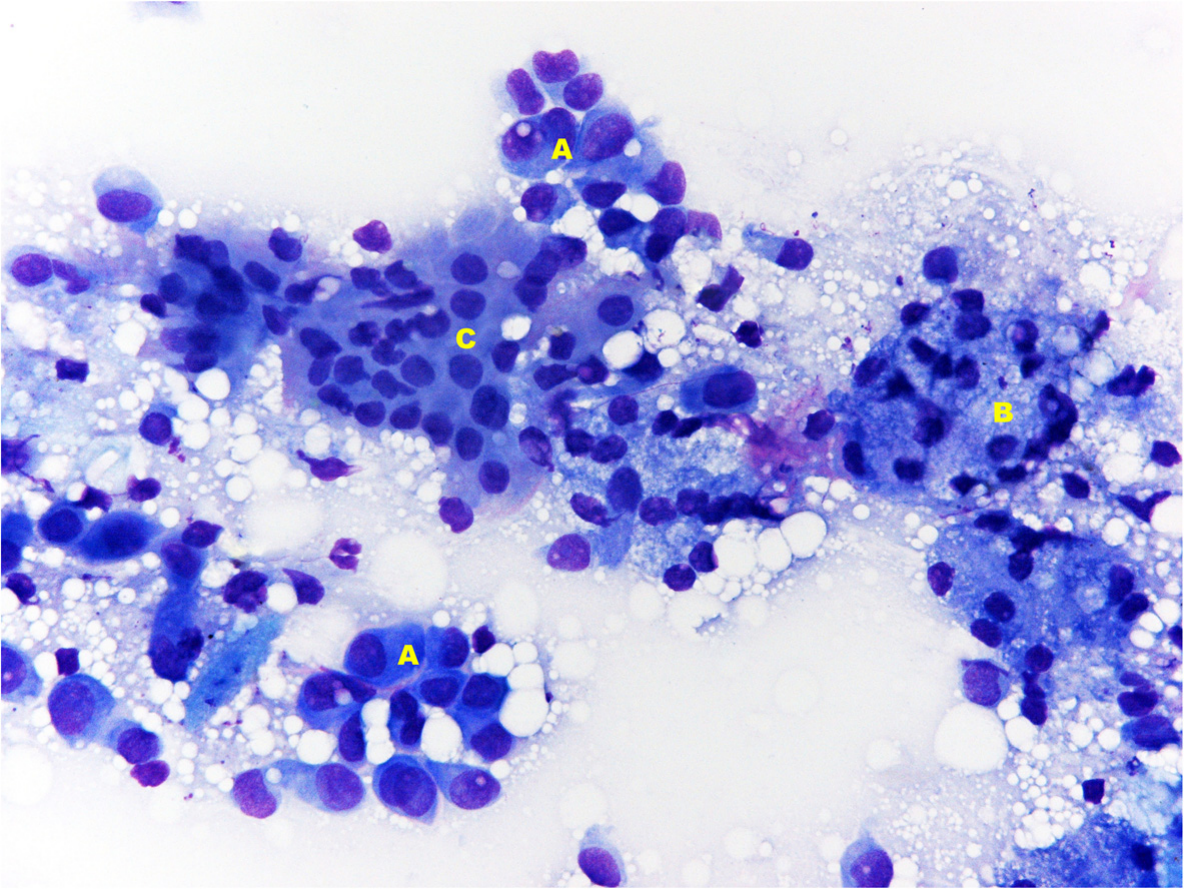
**Fine-needle aspiration biopsy (FNAB) of right submandibular salivary gland shows atypical mesothelial cells with nuclear enlargement and increased chromatin representation (A) and normal salivary gland cells ((B) acinar cells; (C) ductal cells).** May-Grünwald-Giemsa (MGG) x40.

**Figure 3:**
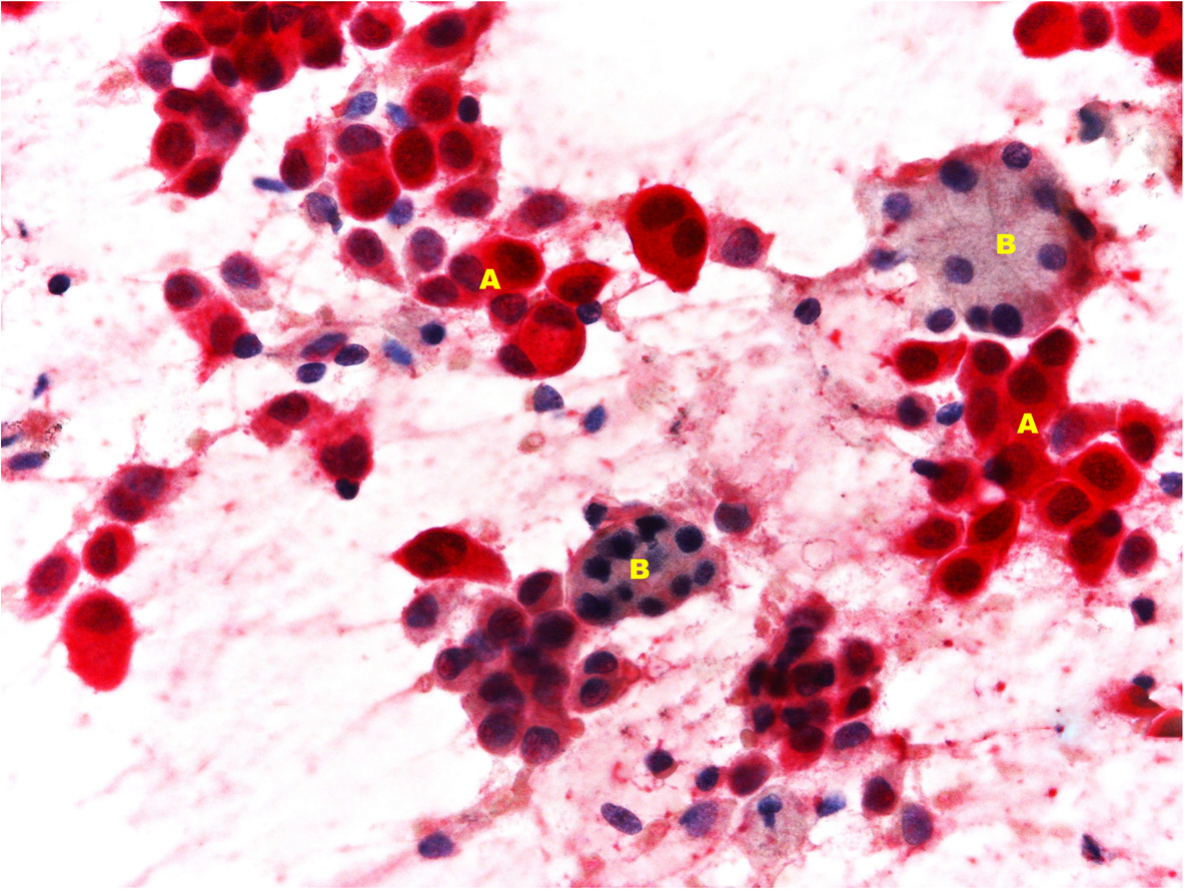
**Fine-needle aspiration biopsy (FNAB) of right submandibular salivary gland shows neoplastic mesothelial cells positive for calretinin (A) and normal acinar glandular cells (B).** Immunocytochemistry, Fast red N.4.

**Figure 4:**
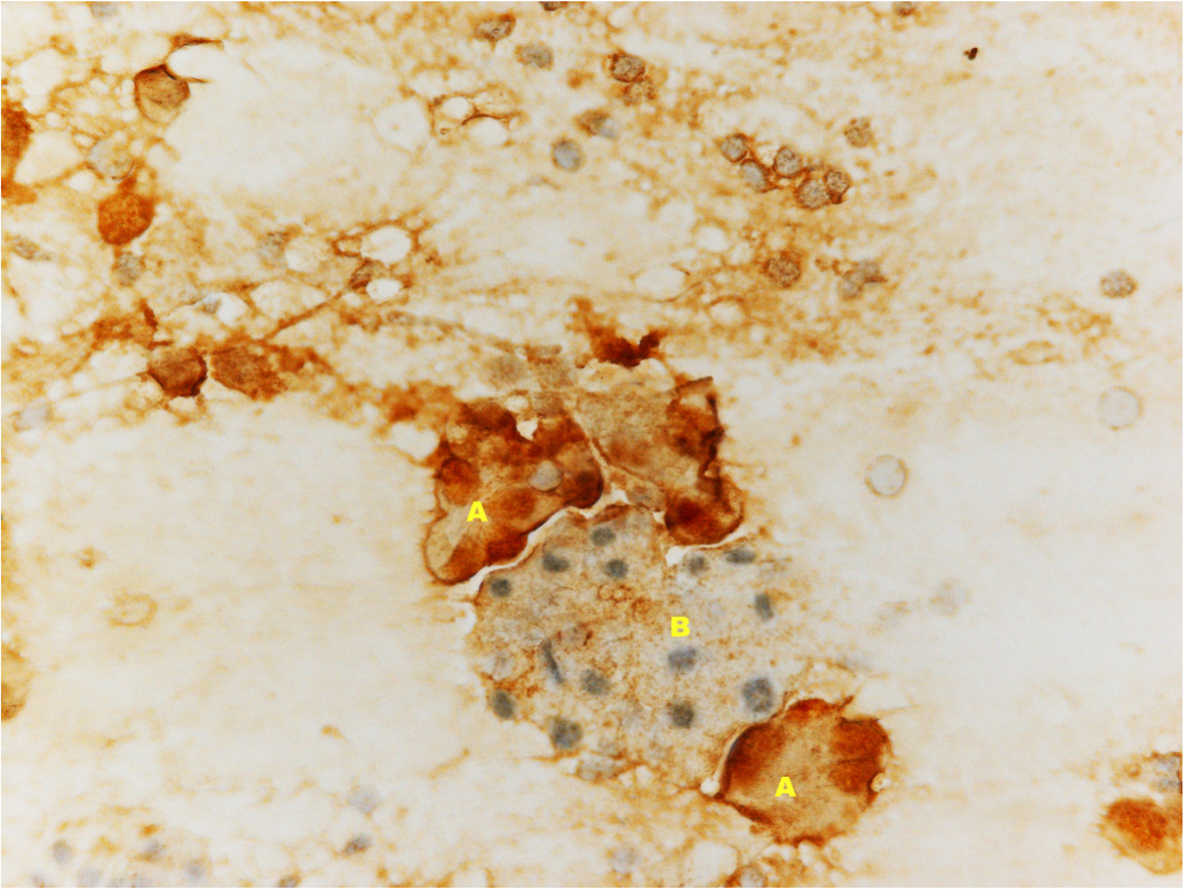
**Fine-needle aspiration biopsy (FNAB) of right submandibular salivary gland, showing nuclear, weak and focal, immunocytochemically WT-1 positive mesothelioma cells (A) and normal acinar salivary gland cells (B).** Immunocytochemistry, DAB.

In November 2012, a right submandibular salivary gland tumefaction was recorded. US examination showed right submandibular gland hypertrophy (Figure 1), confirmed on CT scan and with neither US nor CT signs of metastasis; a diagnosis of inflammation of the gland was made. Anti-inflammatory therapy was administered; the patient also received antibiotic therapy but without improvement. An US-guided FNAB of the right submandibular salivary gland was then performed using a 22-gauge needle and showed atypical mesothelial cells, with nuclear enlargement and increased chromatin representation (Figure 2). Immunocytochemistry showed positivity for calretinin (Figure 3) and nuclear, weak and focal positivity for WT-1 (Figure 4). Based on the previous history of malignant mesothelioma and the morphologic features of the cells, immunocytochemistry allowed confirmation of the diagnosis of right submandibular involvement from epithelioid mesothelioma. The subsequent PET scan was positive for the right submandibular salivary gland. The patient was treated with radiotherapy to this site.

### Literature review

A computerized literature search through MEDLINE, Cancerlit and Embase was performed, in order to make a comparison with our case, applying the words: ‘mesothelioma', ‘metastasis', ‘oral cavity', ‘mouth', ‘buccal', ‘tongue', ‘head and neck area’. Articles and abstracts were also identified by back-referencing from original and relevant papers. Selected for the present review were papers published in English before April 2013.

## Conclusion

With this case report, we would like to focus on two aspects: 1) metastases from mesothelioma to the head and neck area (except for lymph nodes), in particular to the salivary glands, are very rare; 2) diagnosis of metastasis from mesothelioma is made usually by biopsy, and not by FNAB as in our case (Table 1).

**Table 1:** Review of head and neck (except for lymph node) metastases from mesothelioma published in the literature

**Site of metastasis**	**Number of patients**	**Clinical presentation**	**Diagnostic modality**	**References**
Tongue	7	Tongue lesion, nodular consolidation of the tongue with chronic bleeding, submucosal mass, horizontal fissure, swelling of the dorsal surface of the tongue, polypoid lesion	Surgery, lingual incisional and excisional biopsy	[5,7,10,14-16,18]
Mandible	4	Radicular cyst, mandibular gingival mass, periapical radiolucency	Excision of the tooth, biopsy, incisional biopsy, excision of the mass	[5,6,8,12]
Oral mucosa	3	Submucosal mass	Biopsy	[11,17,19]
Lips	1	Lip lesion	Lip biopsy	[9]
Conjunctiva	1	n/a	n/a	[11]
Thyroid	1	n/a	n/a	[11]
Total	17			

We collected the previously published cases of metastases from mesothelioma to the head and neck area, except for lymph node metastases. Tongue was the most frequent site of metastases, with different clinical presentations; no metastases to the salivary glands had previously been found, and no diagnoses of metastases with FNAB had been made.

Reviewing the literature, we found 17 other cases of metastases to the head and neck area (except for lymph nodes) from mesothelioma, but none to the salivary glands. The most involved site was the tongue, with different clinical presentations, followed by the mandible and in particular the mandibular alveolus, and then the oral mucosa. The clinical presentation can be of a benign polypoid-like lesion of the tongue or a radicular cyst in the mandibular alveolus, with only cyto-histological examination allowing a definite diagnosis.

The diagnosis of primary malignant mesothelioma is established by a combination of clinical, imaging, histopathological and immunohistochemical features. The diagnosis of a secondary mesothelioma is easier and can be made more rapidly since there is a previous diagnosis of the primary lesion [19]. However, even with the knowledge of a primary mesothelioma it is necessary to perform a definite diagnosis of a putative lesion to allow appropriate management of the patient.

Reviewing the literature, in almost all cases of head and neck metastases from mesothelioma, incisional or excisional biopsy was performed to obtain the diagnosis; in some cases surgical excision was needed. We found no cases diagnosed by FNAB.

Fine-needle aspiration biopsy (FNAB) is a clinically accepted, minimally invasive technique that allows sampling of tumor for diagnosis by percutaneously directing a needle into the target lesion guided either by direct palpation or under image guidance [20]. In general, the accuracy, specificity and sensitivity of FNAB results depend on the size of the lesion, the method of biopsy, and the histology of the tumor [21]. When FNAB is applied as a diagnostic technique in patients with suspected malignant mesothelioma, the accuracy of diagnosis may be low.

Though the cytologic diagnostic features of malignant mesothelioma were described more than 50 years ago, there is still doubt as to the ability of the cytopathologic modality to obtain a definitive diagnosis of malignant mesothelioma [22-24].

The published sensitivity of cytologic diagnosis of mesothelioma ranges between 32% and 76%, and this wide range of sensitivity is probably related to the sampling technique rather than the interpretation [24].

It must be emphasized that application of immunocytochemical and molecular techniques greatly enhance the diagnostic accuracy of cytologic diagnosis of malignant mesothelioma [25-27]. FNAB biopsies are an ideal method for detecting tumor relapse during the life cycle of the cancer [28], as demonstrated in our case where the imaging techniques of US and CT scans suggested only salivary gland hypertrophy, while FNAB confirmed mesothelioma relapse.

This case is interesting in that it demonstrates a rare site of metastasis from malignant mesothelioma, and shows the efficacy of FNAB for a definite cytologic diagnosis, thus allowing optimum treatment.

## Consent

Written informed consent was obtained from the patient for the publication of this report and any accompanying images.

## Abbreviations

FNAB: fine needle aspiration biopsy; US: ultrasound; CT: computerized tomography; PET: Positron Emission Tomography.

## Competing interests

The authors declare that they have no competing interests.

## Authors’ contributions

MA and EO took care of the clinical case presentation and revised the literature. LC revised the literature and FNAB use, and wrote discussion and conclusion. RPF took care of the pathological aspects and provided cytological figures. All authors read and approved the final manuscript.
